# Correlation between apparent diffusion coefficient values in breast magnetic resonance imaging and prognostic factors of breast invasive ductal carcinoma

**DOI:** 10.1016/j.pbj.0000000000000027

**Published:** 2018-08-03

**Authors:** Ricardo Moutinho-Guilherme, Janeth Hercilia Oyola, David Sanz-Rosa, Israel Thuissard Vassallo, Raquel Murillo García, Joana Martins Pisco, Vicente Martínez de Vega

**Affiliations:** a Department of Clinical Radiology, Hospital Universitario Quirónsalud Madrid; b Department of Biomedical Sciences, Universidad Europea, Laureate International Universities; c Department of Clinical Pathology, Hospital Universitario Quirónsalud Madrid, Madrid, Spain.

**Keywords:** breast ductal carcinoma, breast neoplasms, diffusion magnetic resonance imaging, magnetic resonance imaging, prognosis

## Abstract

**Background:**

We wanted to examine whether the apparent diffusion coefficient values obtained by diffusion-weighted imaging techniques could indicate an early prognostic assessment for patients with Invasive Ductal Carcinoma and, therefore, influence the treatment decision making.

**Objective:**

The main objective was to evaluate the correlation between the apparent diffusion coefficient values obtained by diffusion-weighted imaging and the key prognostic factors in breast invasive ductal carcinoma. Secondary objectives were to analyze the eventual correlations between magnetic resonance imaging findings and prognostic factors in breast cancer; and to perform a comparison between results in 1.5 and 3.0 T scanners.

**Methods:**

Breast magnetic resonance imaging with diffusion-weighted imaging sequence was performed on 100 patients, who were proven histopathologically to have breast invasive ductal carcinoma. We compared the apparent diffusion coefficient values, obtained previous to biopsy, with the main prognostic factors in breast cancer: tumor size, histologic grade, hormonal receptors, Ki67 index, human epidermal growth factor receptor type 2, and axillary lymph node status. The Mann-Whitney *U* test and the Kruskal-Wallis analysis were used to establish these correlations.

**Results:**

The mean apparent diffusion coefficient value was inferior in the estrogen receptor-positive group than in the estrogen receptor-negative group (1.04 vs 1.17 × 10^–3^ mm^2^/s, *P* = .004). Higher histologic grade related to larger tumor size (*P* = .002). We found association between spiculated margins and positive axillary lymph node status [odds ratio = 4.35 (1.49–12.71)]. There were no differences in apparent diffusion coefficient measurements between 1.5 and 3.0 T magnetic resonance imaging scanners (*P* = .513).

**Conclusions:**

Low apparent diffusion coefficient values are related with positive expression of estrogen receptor. Larger tumors and spiculated margins are associated to worse prognosis. Rim enhancement is more frequently observed in estrogen receptor-negative tumors. There are no differences in apparent diffusion coefficient measurements between different magnetic resonance imaging scanners.

**Highlights**

- Magnetic resonance imaging (MRI) with diffusion sequences is helpful in the detection of breast neoplasms.
- Larger tumors and spiculated margins are associated with a worse prognosis.
- Low apparent diffusion coefficient (ADC) values correlate with estrogen receptor (ER)-positive breast invasive ductal carcinoma (IDCs).
- There are no differences in breast ADC measurements between different MRI scanners.

## Introduction

Breast cancer is the most frequently diagnosed cancer and the leading cause of cancer death among females worldwide.^1^ Invasive ductal carcinoma (IDC) is the most common histologic type of breast cancer, accounting for approximately 75% of all cases.^2,3^ In spite of the advances in the detection of breast cancer with mammography and ultrasound, differentiating benign and malignant lesions still represent a difficult diagnostic problem, particularly in dense fibroglandular breasts. Several studies have been executed to determine the diagnostic performance of contrast-enhanced magnetic resonance imaging (MRI) in breast lesions.^4,5^ This procedure has demonstrated a sensitivity of 94% to 100% in the detection of breast cancer.^6^ Specificity has, however, generally been lower and more variable, ranging from 37% to 97%.^7^ This is caused by the false positives such as hormonal therapy, the menstrual cycle, fibroadenomas, papillomas, or proliferative alterations.

Diffusion-weighted imaging (DWI) is a noninvasive technique that represents the microscopic random movements of the hydrogen protons, contained in water molecules, in the interstitial (extracellular) space. This MRI sequence provides information about the biophysical characteristics of a tissue, mainly about its cellularity. It is possible to quantify these proton movements by calculating the apparent diffusion coefficient (ADC), which measures the water molecules displacement in mm^2^ per second.^8^ In high cellularity tissues, for example, in malignant tumors with abundant mitosis, the movement of water molecules in the interstitial space is restricted and the ADC values are low. On the contrary, in low cellularity tissues, like the normal fibroglandular tissue or benign tumors, the water molecules move freely in the interstitial space increasing ADC values. ADC values are also altered by fluid viscosity, membrane permeability, and blood flow. The ADC is calculated for each pixel of the image and is displayed as a parametric map. A region of interest (ROI) is drawn on the map, so the ADC can be derived on different tissues.^9^

DWI is the primary modality used to evaluate acute cerebral infarction. It has been extensively applied to assess other organs such as the liver, pancreas, ovaries, prostate, and, most recently, the breast.^10–16^ Nowadays, it has extended its use to multiple fields in oncology.^10^ DWI measurements usually take 2 to 5 minutes to perform, and there's no need to administrate any kind of exogenous contrast medium.

Several articles have shown that the DWI is useful to characterize breast lesions and, therefore, a very helpful technique to differentiate between benign lesions (high ADC values) and malignant tumors (low ADC values) in this organ.^10^ These studies used different *b* values, ranging from 0 to 1000 s/mm^2^, and found a significant difference in ADC values between malignant and benign lesions, with a sensitivity varying from 81% to 93% and a specificity from 80% to 88%, for an ADC cut-off of 1.10 up to 1.30 × 10–3 mm^2^/s. On MRI scanners, diffusion sensitivity is easily altered by changing the *b* value parameter, which is mainly proportional to the gradient amplitude and duration.^9^

Classic prognostic markers in breast cancer include tumor size, histologic grade (HG), estrogen receptor (ER), and progesterone receptor (PR), Ki67 proliferation index, human epidermal growth factor receptor type 2 (HER2) protein and axillary lymph node (N) status. In addition, these factors greatly influence the choice of surgical procedure and the decision to administer neoadjuvant chemotherapy.^17^ Until now, there have been few studies about using DWI to establish a solid correlation between these prognostic factors and the ADC values in malignant breast tumors.^18–20^

The purpose of the present study is to compare the ADC values of DWI previous to biopsy in 100 cases of breast IDC with the main prognostic factors in breast cancer: tumor size, HG, hormonal receptors (ER and PR), Ki67 index, HER2, and N status.

Secondary objectives were to analyze the correlations between MRI findings (margins of the lesion, internal enhancement, and kinetic curve type) and the same prognostic factors in breast cancer; and to perform a comparison between the ADC measurements in 1.5 and 3.0 T MRI scanners, to detect any significant differences.

## Methods

### Patients

Our institutional clinical committee approved the retrospective design and renounced written informed consent. We enrolled 308 consecutive patients who underwent breast MRI with diffusion sequence in our institution between November 2016 and June 2017, before the breast biopsy for the suspicious mass. Inclusion and exclusion criteria were very extensive and rigid to avoid biases as maximum as possible. Cases involving previous excisional breast biopsy (n = 7); any type of previous breast surgery (n = 35); previous detection/treatment of other suspicious breast lesions (n = 37); no pathological confirmation of the breast biopsy (n = 6); breast neoadjuvant chemotherapy, endocrine therapy, or radiotherapy (n = 14); other types of malignant breast tumors (n = 41); nonmass type lesions of the breast (n = 20); bilateral breast lesions (n = 9); benign breast lesions (n = 35); and male patients (n = 1) were excluded. Three lesions were excluded because of technical issues (DWI acquisition and failure of lesion detection). As a result, we included 100 female patients with 100 breast cancers, all histopathologically confirmed to be IDC, in this study. We give special emphasis to the fact that the patients included in this study had not performed any breast biopsy previous to the DWI, for this technique may result in a change of the ADC value disrupting the tumor architecture and creating edema. Furthermore, the MRI with diffusion sequence is performed before the biopsy at our institution as standard protocol, so that the determination of the ADC values is not influenced by the pre-existing knowledge of what type of lesion the patient presents.

### Breast MRI

The MRI were acquired with two 1.5 T scanners (Signa HDxT and Optima 450w GE Healthcare, Milwaukee, WI) and one 3.0 T scanner (Signa HDxT) all of them equipped with a dedicated 8 channel breast coil. MRI was performed using the following sequences: sagittal fast spin-echo T2-weighted; axial DWIs of both breasts were obtained at *b* values of 0 and 700 s/mm^2^ with the parameters: repetition time (TR) 6000 (3.0 T) to 7100 (1.5 T) ms; echo time (TE) 51.0 ms; number of excitations: 7 (3.0 T) to 10 (1.5T); flip angle 90°; field of view (FOV) 320 × 320 mm; matrix 128 × 128; slice thickness 3.0 mm; no gap. Fat suppression was applied using a spectral attenuated inversion recovery technique. Dynamic contrast-enhanced-MRI were obtained by a 3D Vibrant sequence on the sagittal plane with the following parameters: TR 4.6 (3.0 T) to 6.1 (1.5 T) ms, TE minimum, flip angle 10°, slice thickness 2.6 (3.0 T) to 3 (1.5 T) mm, matrix 320 × 224, FOV 20 cm, and temporal resolution range of 72 (3.0 T) to 80 (1.5 T) seconds. Acquisition was performed before and 5 times after intravenous administration of 0.2 mL/kg of gadolinium chelates at a rate of 2.0 mL/s (Magnevist, Bayer-Schering, Berlin, Germany), followed by a saline flush. Finally, an axial 3D vibrant sequence of both breasts was acquired (delayed contrast sequence) with the following parameters: TR 4.6 (3.0 T) to 6.1 (1.5 T) ms, TE minimum, flip angle 10°, slice thickness 3 mm, matrix 384 × 320, FOV 32 cm, and temporal resolution range of 40 (3.0 T) to 75 (1.5 T) seconds.

Images were assigned to a workstation (Advantage Window 4.4; GE Medical System, Milwaukee, WI) for processing. Parametric quantitative ADC maps were generated by Functool software (GE Healthcare). ADC was obtained by placing ROIs, within the margins of the primary lesions on the ADC maps, and calculated according to the following equation: ADC = −(1/*b*) ln (*S*/*S*_0_), where *b* is the diffusion factor and *S*_0_ and *S* are the signal intensities in ROIs obtained with different gradient factors (*b* values of 0 and 700 s/mm^2^, respectively).

**Figure 1:**
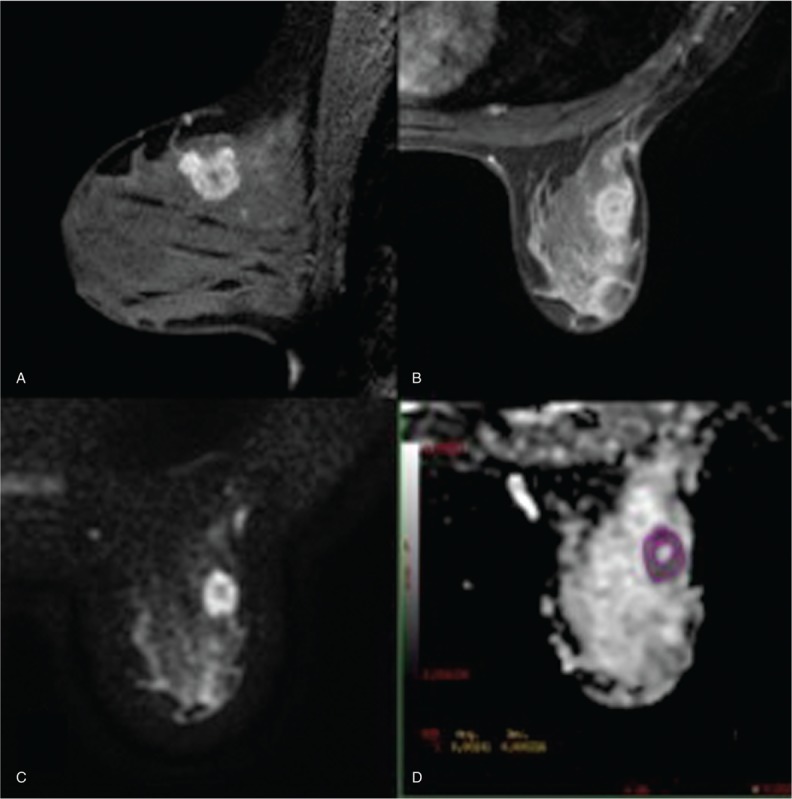
Thirty-eight-year-old woman with invasive ductal carcinoma in the upper external quadrant of the left breast. A, Sagittal dynamic contrast-enhanced magnetic resonance imaging 2 minutes after contrast enhancement identified a round mass with subtle irregular margins. The lesion was characterized by peripheral enhancement with a small central hypovascular area. B, Axial dynamic contrast-enhanced magnetic resonance imaging 10 minutes after contrast enhancement (delayed phase). C, On diffusion-weighted images acquired with a *b* value of 700 s/mm^2^, the tumor appeared as a hyperintense mass with a central hypointense component. D, A region of interest (ROI) within the margins of the lesion was manually traced on apparent diffusion coefficient maps, avoiding the central part. Apparent diffusion coefficient value was equal to 1.01 × 10^–3^ mm^2^/s. At histopathology, the lesion was defined as a grade III invasive ductal carcinoma. At the immunohistochemical analysis, hormonal receptor status was positive (estrogen and progesterone receptors), Ki67 expression was 60% and human epidermal growth factor receptor type 2 (HER2) status was equal to 0 (negative). Axillary lymph node status was negative.

The ROI was manually drawn in the slice in which the cancer presented a greater diameter (Fig. 1). Apparent necrotic or cystic components were avoided by referring to other MRIs. In cases with multifocal or multicentric masses the largest tumor was selected. The image analysis was performed by 2 radiologists with 2 and 20 years of experience on breast MRI. The final ADC value for each patient was obtained by consensus between the 2 radiologists, with the lowest being the preferred number.

The lesions were assessed for size, margins, and internal enhancement pattern. The evaluation of kinetic curve (type 1, 2, or 3) was performed. All characteristics were defined based on the breast imaging-reporting and data system classification.

### Histopathological analysis

All tumors were biopsied after performing breast MRI. Percutaneous biopsies were guided with ultrasound (14 G core biopsy) or MRI (9 G vacuum-assisted biopsy).

All patients underwent surgery for the breast mass at our institution and lymph node dissection was performed on those with positive axillary findings. The final diagnosis for IDC was made with histopathological examination. All pathological slices were evaluated by 2 pathologists, with years of experience in breast pathology, and the results were presented by consensus. Pathologic reports were reviewed.

Firstly, the longest diameter of the tumor was measured from the gross specimen. Then, the samples of the tissue were fixed in 10% buffered formalin and embedded in paraffin. Five-micrometer sections were stained with hematoxylin-eosin. The HG was assessed using the Nottingham modification of the Bloom-Richardson system (Elson-Ellis method) considering tubular formation, nuclear pleomorphism, and mitotic count (each one scored from 1 to 3 points). Mitotic figures were only counted at the periphery of the tumor in the most mitotically active area. Scoring was performed (3–5 points was considered grade I, 6–7 was grade II, and 8–9 was grade III). Lymph node specimens were obtained by sentinel N resection followed by immediate dissection if 1 or more were positive in preoperative examination. The N status (positive/negative) was assessed histologically on routinely stained sections. The presence of 1 metastasis was considered as a positive finding. Macrometastases were defined as the presence of at least 1 positive axillary lymph node with a larger diameter of >2 mm, whereas a diameter of ≤2 mm was marked as micrometastasis.

In addition, ER, PR, HER2, and Ki67 were analyzed as molecular prognostic markers. An immunohistochemical analysis was performed using commercially available antibodies (Master Diagnóstica, Granada, Spain).

Hormone receptors (ER and PR) were evaluated using Quick (Allred) Score (QS) that measures the reactivity from 0 to 8 points adding 2 values: the percentage of positive cells (0–5) and the intensity of the staining (0–3). ER and PR positivity were defined as scores of 1 point or more.

The HER2 expression was determined immunohistochemically and classified as 0, 1+, 2+, or 3+ based on the Sociedad Española de Anatomía Patológica (Spanish Society for Anatomical Pathology) and College of American Pathologists guidelines: 0 for no immunoreactivity or immunoreactivity in ≤10% of tumor cells, 1+ for faint weak immunoreactivity in >10% of tumor cells and only a portion of the membrane was positive, 2+ for weak to moderate complete membrane immunoreactivity in >10% of tumor cells or circumferential intense membrane staining in ≤30% of cells, and 3+ if >30% of the tumor cells showed circumferential intense and uniform membrane staining. Scores of 0 and 1+ were considered as negative for overexpression of HER2, score 2+ was regarded as equivocal and fluorescent in situ hybridization was performed and 3+ was considered positive.

For Ki67, the staining property in tumor cells was expressed as a percentile value (%) with Mib-1 monoclonal antibody (1:200 dilution; Dako, Glostrup, Denmark). Ki67 staining of ≥20% was considered positive expression and <20% was considered negative expression.

### Statistical analysis

The Kolmogorov-Smirnov test was performed to determine normality of data distribution. Parametric variables are expressed as mean and standard deviation, and nonparametric variables as median and interquartile range for each prognostic factor (tumor size, HG, ER, PR, Ki67, HER2, and N status). The data analysis was performed to evaluate statistically significant differences. Odds ratios (ORs) are expressed along with 95% confidence intervals.

Differences on ADC values between 2 groups were performed by Student *t* test analysis. One-way analysis of variance test was performed to test the difference between more than 2 groups, followed by post-hoc Honest Significant Difference Tukey test. For variables with nonparametric distribution, the Mann-Whitney *U* test or Kruskal-Wallis analysis were used. The Pearson correlation test was used to study the relationship between the ADC values of the IDC with pathological prognostic parameters, as well as to correlate the tumor grade with tumor size.

The correlation coefficient *r* and *P* value were calculated. The 2-tailed *P* value was considered statistically significant if *P* ≤ .05 at a confidence interval of 95%. The statistical data analysis was performed by using the software program SPSS (Statistical Package for Social Science, version 21.0, Chicago, IL).

## Results

The ADC value was measured and histologic results were reviewed for 100 patients, in whom IDC was detected on DWI.

### ADC value analysis

The mean ADC value of IDC was 1.06 ± 0.18 × 10^–3^ mm^2^/s. The minimum, maximum, and mean ADC values with standard deviationSD according to the pathological prognostic factors are represented in Table 1.

**Table 1:**
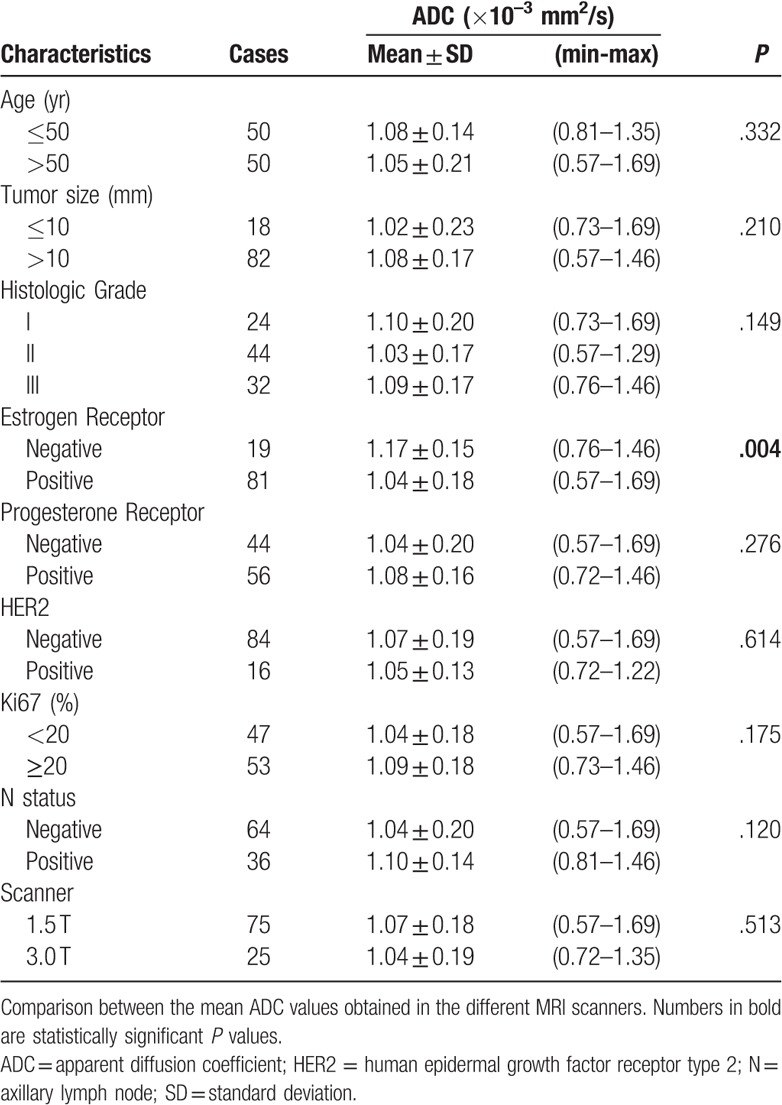
Correlation between apparent diffusion coefficient values and prognostic factors

All patients were women and aged 25 to 82 years old (mean age of 52.3 ± 12.1 years old). There were no differences between ADC values in older and younger patients (>50 vs ≤50 years old).

The median size of breast IDC was 17.00 mm with an interquartile range of 11.00 mm (mean = 19.06 mm). The mean ADC value of IDC ≤10 mm (n = 18) was 1.02 ± 0.23 × 10^–3^ mm^2^/s and of >10 mm (n = 82) was 1.08 ± 0.17 × 10^–3^ mm^2^/s. The ADC values between larger and smaller tumors were not significantly different.

The mean ADC value of grade I (n = 24) was 1.10 ± 0.20 × 10^–3^ mm^2^/s, of grade II (n = 44) was 1.03 ± 0.17 × 10^–3^ mm^2^/s, and of grade III (n = 32) was 1.09 ± 0.17 × 10^–3^ mm^2^/s. There were no significant differences in ADC values of higher grade compared to lower grade tumors.

Eighty-one tumors were ER positive. Fifty-six percent of all cases were PR positive. Only ER expression presented a statistically significant negative correlation with ADC values (*P* = .004). The mean ADC value was lower in the ER-positive group (1.04 ± 0.18 × 10^–3^ mm^2^/s) than in the ER-negative group (1.17 ± 0.15 × 10^–3^ mm^2^/s). No statistically significant correlation was found between PR expression and ADC values.

There was no significant relationship between mean ADC values and HER2 expression. Six percent of all patients presented HER2-positive staining.

Positive Ki67 expression occurred in 53 biopsies. This molecular prognostic marker had no statistically significant correlation between higher and lower ADC values.

Micrometastases were detected in 12 patients, whereas 24 presented macrometastasis. The remaining cases (n = 64) had negative axillary findings. Patients with negative N metastasis presented similar ADC values of IDC compared to patients with positive N status (1.04 ± 0.20 × 10^–3^ vs 1.10 ± 0.14 × 10^–3^ mm^2^/s; *P* = .120).

In addition, association between tumor size and traditional prognostic factors was analyzed. HG showed a positive significant correlation with tumor size. By calculating the proportions of each HG in relation to tumor size, we found that a higher HG related to a larger tumor size (Table 2). Approximately 93.7% of HG III tumors were larger than 10 mm, whereas only 58.3% of HG I tumors were larger than 10 mm. There was no statistical correlation between tumor size and N status.

**Table 2:**
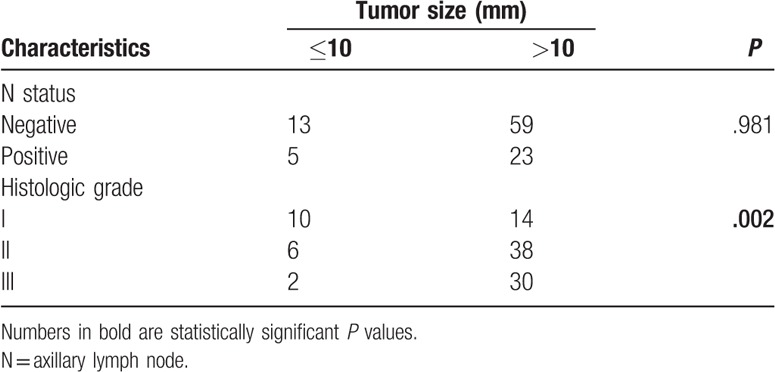
Correlation analysis between traditional prognostic factors

### Imaging analysis

All of the 100 cases analyzed presented mass type lesions (nonmass types were excluded from the study). Thirty-seven lesions were located on the right breast and 63 on the left one. Bilateral cases were excluded. The most frequent location was on the external upper quadrant (39%) and there was a lesion occupying the totality of one breast (sized 50 cm). Fifty-two percent of IDC were unifocal and 48% multifocal.

Sixty out of the 100 cases presented spiculated margins and 40 showed irregular margins. There were 20 lesions presenting rim enhancement, 63 heterogeneous enhancement, and 17 homogeneous enhancement. The kinetic curve types were persistently enhancing (type 1, progressive) in 14%, plateau (type 2) in 28%, and washout (type 3) in 58% lesions.

In addition, we performed an analysis on the potential relationships between breast cancer prognostic factors and MRI findings, which could be of clinical importance. We found a solid and significant association between spiculated margins and positive N status with an OR of 4.35 (1.49–12.71).

Interestingly, there was also an association between spiculated margins and positive ER findings with an OR of 3.25 (1.15–9.17); it is 3 times more probable that a spiculated margin relates to a positive ER finding than an irregular margin.

Finally, there was an association between rim enhancement and ER-negative subgroups; with an OR of 7.89 (2.58–24.13), it is more likely that a rim enhancement relates to a negative ER than a homogeneous/heterogeneous enhancement.

Table 3 summarizes the associations between the MRI findings and the different prognostic factors.

**Table 3:**
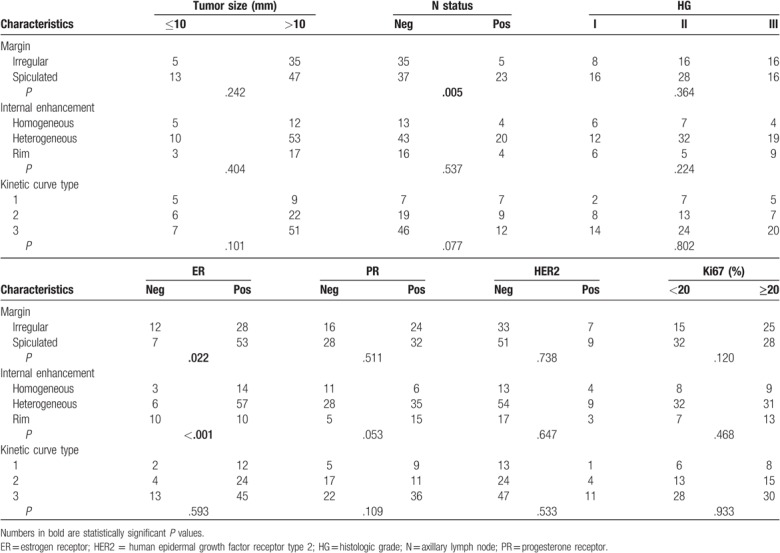
Correlation between magnetic resonance imaging findings and traditional prognostic factors

Because of the different MRI scanners used in this study, we assessed the variability of the mean ADC values obtained with each magnet (Table 1). We found no significant differences of the values among the different scanners.

## Discussion

Our main goal was to examine whether the ADC values obtained by DWI techniques could indicate an early prognostic assessment for patients with IDC and, therefore, influence on the treatment decision making. We found that low ADC values correlate with ER-positive groups. Moreover, it is more likely that a spiculated margin is associated with a positive N status and positive ER than an irregular margin. A rim enhancement was more associated with a negative ER than a homo/heterogeneous enhancement. In the present study, we found no statistically significant differences in ADC measurements between 1.5 and 3.0 T MRI scanners.

IDC has a more fustigate prognosis than other histopathological types of breast cancer. Lower ADC values for this specific subtype have been reported in several studies.^21,22^ This can be explained by the tightly packed tumor cells which constrain the water molecule's motion and limit diffusion. In our study we found an overall ADC for IDC of 1.06 ± 0.18 × 10^–3^ mm^2^/s. In this sense, Marini et al^23^ reported a cut-off of 1.10 × 10^–3^ mm^2^/s, with 80% sensitivity and 81% specificity to distinguish between malignant and benign breast lesions.

We evaluated the possible correlations between the main prognostic factors in breast IDC (tumor size, HG, ER, PR, Ki67 index, and N status) and the ADC values. ER was the solely biomarker that demonstrated a significant difference in the mean ADC values. The ADC was significantly lower in the ER-positive group. Similar results have been previously reported.^19,20,24^ It has been presented that ER expression is related with inhibition of angiogenesis and decreased perfusion, and it is also related with a favorable prognosis.^25,26^ Therefore, it is plausible to consider that the ADC values were significantly lower in the ER-positive group due to the smaller role of perfusion produced by angiogenesis compared to the ER-negative group.

Furthermore, highly proliferating tumors with high HG (III) were related to larger measurements in the MRI. Grade III tumors were more likely to be >10 mm size. Considering that the number of mitosis reflects the tumor HG, which is an image of its cellularity, it is conceivable to hypothesize the motive why IDCs characterized by high cellularity (or a higher number of mitosis) show the lower ADC values at DWI. In fact, high-grade tumors have been related with lower ADC values in various malignancies, including hepatocellular carcinoma, pancreatic cancer, and clear cell renal carcinoma, among others.^27–29^ The relationship between larger tumor sizes and reduced survival rates for breast cancer patients has been established. Tumors with greater sizes typically have more probability of metastatic disease, and for this reason, they are connected to a worse prognosis.^21,22^ Nevertheless, there are exceptions as in the case of the triple negative breast cancers (from 12% up to 24% of all cases), which do not have an exact relation between tumor size or N status and prognostic outcome.^30,31^

To date, the association between the MRI findings and the histology is a matter of controversy. In this sense, we found that spiculated margins are a risk factor for positive N status and ER-positive findings. In our study, we could infer that it is 4 times more probable that a spiculated margin relates to a positive N status than an irregular margin. Previous studies have found that a spiculated margin has a positive predictive value for malignancy, ranging from 76% to 88%.^32,33^ Spiculated margins have also been associated with lower HG and positive PR expression.^32^ In contrast, other studies have reported that spiculated margins were related to negative ER and positive HER2 subgroups.^34,35^ Wang et al^35^ concluded that ER-positive groups show spiculated margins with no relation with its HER2 expression.

Rim enhancement is an essential morphologic sign in the differential diagnosis of breast lesions. In our study, there was a correlation between rim enhancement and ER-negative tumors. Rim enhancement is a result of the increased angiogenic factors in the periphery of the tumor along with central necrosis and fibrotic areas. Numerous studies have demonstrated rim enhancement correlations with greater tumor sizes; greater HG, ER, and PR-negative expressions; greater Ki67 index; and positive N status.^33,34^ They concluded that rim enhancement is more commonly observed in rapidly growing carcinomas.

One of the proposed objectives was to study whether the ADC measurements on the different MRI scanners were significantly different or not. We observed no differences between the 1.5 and 3.0 T scanners. Opposite to our findings, Jeh et al^24^ reported higher ADC values in a 3.0 T scanner than in a 1.5 T scanner. However, the different magnetic fields and *b* values used between the MRI scanners may influence the ADC measurements, as they can interfere with diffusion sensitivity. Pereira et al^36^ concluded that using lower *b* values had connection to higher ADC measurements and vice versa, adding that the ADC calculated from *b* values of 0 and 750 s/mm^2^ (as in our study) was marginally better than any other combinations.

We found no statistical significance relating the mean ADC value and the tumor grading. Yoshikawa et al^37^ reported that breast cancer cellularity has no relation with the ADC values. Another study^20^ added that the median ADC value was 1.11 × 10^–3^ mm^2^/s in HG I tumors, 1.09 × 10^–3^ mm^2^/s in HG II tumors, and 1.06 × 10^–3^ mm^2^/s in HG III tumors, but did not found a statistical significance as well (*P* = .82). Jeh et al^24^ did not find any relation between HG and ADC values. Additional analyses are required to assess the clinical usefulness of the ADC values in tumor grading.

Other prognostic factors, including tumor size, PR, HER2, Ki67, and N status showed no significant correlation with ADC values. Kim et al^20^ reported similar findings, with a marginal significance (*P* = .053) in the relationship between lower ADC values and ER-positive patients.

It has been recently published^38^ that lower ADC values relate to the presence of N metastasis. This can be explained by the difference in study populations, because tumor size is a risk factor for N positive status. The referred study involved many large cancers (>50 mm), whereas in our study the smallest lesion analyzed was 6 mm in size and the largest 50 mm.

In this study, we did not analyze cancer recurrence or survival rate, only the referred prognostic factors. Further study by long-term follow-up is necessary to clarify prognoses. Despite being placed in several different places, we used a relative small-sized ROI for ADC measurements, and it may not entirely reflect all of the tumor characteristics. Patient movement during the acquisition of the MRI could lead to incorrect ADC measurements. Our retrospective design could be a limitation as well.

## Conclusions and considerations for the clinical practice and future investigation

As there is great disparity about these correlations in the present time, we carried out a basic analysis of the existing literature about this subject. Other important studies with a similar design and objectives were analyzed to compare the various results with our findings. We did not intend to perform an extensive meta-analysis on the subject. All of the studies intended to correlate the ADC values obtained in DWI techniques with any of the prognostic factors in breast cancers. The conclusions of each study that we reviewed are summarized in Table 4.

**Table 4:**
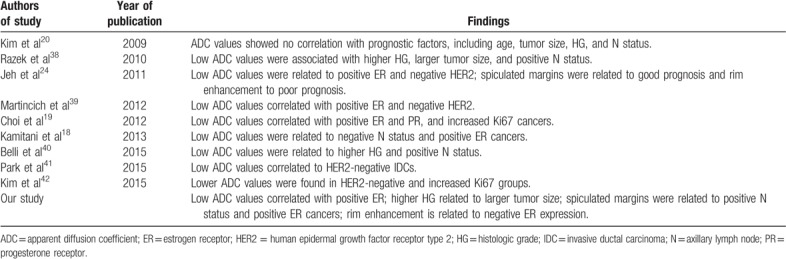
Comparison between previous studies on the same subject

There are obvious points of discordance between these studies. Many of them studied ADC values on several types of breast cancers, including benign forms. Others used distinctive types of measurements on ADC values and analyzed other variables. Different sizes of populations were selected and not always in a homogeneous way. Also, the ROI used in each study may vary significantly, which emphasizes the possible different interobserver measures in ADC values. These possible differences have been studied before^43,44^ and there has been evidence that different methods of ROI demarcation (the whole lesion vs a small fixed area) may influence ADC values.

Emphasis must be given to the fact that, in our study, previously biopsied breasts were excluded. This is the main point that differentiates this study from the preceding ones. In our experience, biopsy can cause disruption of tumor architecture and formation of edema resulting in a change of the ADC value. DWI techniques performed after biopsies were therefore excluded in our investigation. None of the studies we analyzed makes any reference to this exclusion criteria, which could lead to an important source of bias. Further study on this matter is needed to investigate the possible differences between ADC values previously and after biopsy procedures.

In addition, MRI diffusion sequences have been acknowledged as potential biomarkers for anticancer therapy response.^45^ In this scenery, both baseline ADC values and changes during treatment have been related with tumor response.^46,47^

The highly different conclusions between these articles, reinforces our opinion that further study on this matter is required to achieve higher levels of concordance in results. Independently of the present findings, we consider that MRI techniques with DWI sequence represent an important role on early detection and prognostic evaluation of breast cancer, especially in IDC types, and can be easily implemented into standard clinical protocols.

In conclusion, we found that low ADC values are a common finding in IDCs with positive expression of ER. Larger tumors and spiculated margins were associated to a worse prognosis. Rim enhancement is more frequently observed in ER-negative tumors. Clinically, there are no differences in breast ADC measurements between different MRI scanners (1.5 and 3.0 T).

## Acknowledgments

The authors would like to thank the Hospital Universitario Quirónsalud Madrid MRI technicians for the high-quality images acquisition and all the Clinical Radiology department members for their help in the elaboration of this study.

### Conflicts of interest

The authors declare that they have no competing interests in this study. All authors read and approved the final manuscript.
